# Choline Chloride Based Natural Deep Eutectic Solvents as Extraction Media for Extracting Phenolic Compounds from Chokeberry (*Aronia melanocarpa*)

**DOI:** 10.3390/molecules25071619

**Published:** 2020-04-01

**Authors:** Maša Islamčević Razboršek, Milena Ivanović, Peter Krajnc, Mitja Kolar

**Affiliations:** 1 University of Maribor, Faculty of Chemistry and Chemical Engineering, 2000 Maribor, Slovenia; masa.islamcevic@um.si (M.I.R.); milena.ivanovic@um.si (M.I.); peter.krajnc@um.si (P.K.); 2 University of Ljubljana, Faculty of Chemistry and Chemical Technology, 1000 Ljubljana, Slovenia

**Keywords:** chokeberries (*Aronia melanocarpa*), phenolic compounds, green solvents, natural deep eutectic solvents, ultrasound-assisted extraction, HPLC

## Abstract

For the isolation of selected phenolic compounds from dried chokeberries, natural deep eutectic solvents (NADESs) were investigated as a green alternative to conventionally used extraction solvents. Four types of NADESs were synthesised, with choline chloride as the hydrogen bond acceptor in combination with different hydrogen bond donors (sugars, organic acid and urea). Ultrasound-assisted extraction was used to improve the extractability of the phenolic compounds and the results were compared to those obtained with 80% methanol as the extraction media. The highest values of total phenols and total flavonoids were found in the extract obtained with choline chloride–fructose NADES (36.15 ± 3.39 mg gallic acid g^−1^ dry weight (DW) and 4.71 ± 0.33 mg rutin g^−1^ DW, respectively). The extraction recoveries for the individual phenolic compounds depended strongly on the phenolic compound’s structure, with relative mean values between 70% and 97%.

## 1. Introduction

The growing interest in a healthy lifestyle and maintaining a sustainable environment means an increasing need to find natural antioxidants to replace synthetic compounds. Consequently, many researchers have focused on the investigation of bioactive compounds (especially those from the polyphenol classes) from various plant materials [1,2]. Recent studies have shown that berries contain the highest number of phenolic compounds and have the highest antioxidant activity among other fruits [3,4,5,6].

Black chokeberry (*Aronia melanocarpa*) belongs to the *Rosaceae* family. It is widespread in North America and has also become common in Europe [5]. Due to the astringent, sour and bitter taste of fresh chokeberry, this fruit is mainly processed in the food industry into juices, jams, fruit teas and food supplements [7]. From a phytochemical point of view, chokeberry is documented as one of the richest sources of bioactive phenolic compounds, such as anthocyanins, proanthocyanidins and phenolic acids [8,9]. There is strong scientific evidence for the health benefits of chokeberry [10,11,12]. As reported, the positive effect of chokeberry consumption is reflected in its ability to prevent and treat various diseases such as colon cancer [13], high blood pressure [14], and high levels of low-density lipoprotein cholesterol [8], among others.

For the extraction and separation, various extraction methods were previously developed/studied within our group [15,16,17,18]. Likewise, various extraction methods using organic solvents have been developed and published for the extraction and separation of chokeberry phenolic compounds, including the traditional maceration method [19], ultrasound-assisted extraction (UAE) [4] and microwave-assisted extraction (MAE) [9].

However, new trends in chemistry and environmental protection requirements have led to increased interest in the use of non-toxic, low-cost and biodegradable green solvents. So far, ionic liquids (ILs), as the first represented green solvents, have attracted considerable attention of scientists from different research fields [20]. Nevertheless, in contrast to their primarily green character, some authors have pointed out a significant number of shortcomings that limit the use of ILs in large-scale processes, including their generally low biodegradability and high treatment costs [21]. Consequently, a newly formed subclass of ILs consisting of hydrogen bond acceptors (HBAs) and hydrogen bond donors (HBDs), called “Deep Eutectic Solvents” (DESs), was first described by Abbott et al. in 2003 [22]. Typically, DESs consist of two or more solid organic or inorganic compounds that form a stable liquid after mixing. Under optimal conditions (temperature and stirring time), an eutectic mixture with a melting point lower than that of the compounds that make up the mixture is formed [23]. Otherwise, if the eutectic mixture consists of metabolites naturally present in all types of cells and organisms, these newly formed systems are called “Natural Deep Eutectic Solvents” (NADESs) [24]. DESs have already shown applications in various fields, including organic synthesis [25], preparation of inorganic materials [26], and biochemistry [27]. Currently, the use of DESs/NADESs in separation processes is rapidly increasing, especially in the extraction of phenolic compounds from plants [28,29,30,31,32,33,34]. DESs/NADESs have important advantages over conventionally used organic solvents, such as no or low toxicity, biodegradability, low cost and easy preparation [24]. While the efficiency of classical extraction processes can be significantly improved by applying UAE [35,36], a combination of UAE and NADESs can be also effective [33,37,38,39].

Therefore, this work focuses on the synthesis of hydrophilic NADESs and the determination of their potential in the extraction of chokeberry phenolic compounds. This is the first report in which bioactive compounds from chokeberry have been extracted with NADESs. Consequently, the results obtained were compared with those obtained with the most commonly used UAE-methanolic extraction. Furthermore, the optimization and validation of the high-performance liquid chromatography with diode-array detector (HPLC-DAD) method for the simultaneous identification and quantitative determination of 21 phenolic compounds from different sub-classes in a single chromatographic run was performed.

## 2. Results and Discussion

### 2.1. Preparation of NADESs

In general, the extraction capacity for different classes of phenolic compounds in plant extracts can potentially be influenced by both the extraction conditions applied (extraction technique, temperature, solvent/liquid ratio) and by the physicochemical properties of the solvent used. Similarly, the chemical composition of a tailor-made NADES is crucial for its extraction capacity for phenolic compounds, as it determines properties such as polarity, physicochemical interactions, solubility and viscosity [40,41]. Consequently, with the aim to select the most appropriate NADES for the extraction of chokeberry polyphenols, three groups of HBDs (amide (urea), organic acid (lactic acid) and two carbohydrates-sugars (fructose and glucose)) were used in combination with choline chloride as the HBA to prepare NADESs (Table 1). The initial synthesis of NADESs based on sugars (fructose and glucose) was not possible without the addition of water.

All prepared solvents proved to be stable, transparent and highly viscous liquids that do not form precipitates during preparation, extraction and analysis. Most of the NADESs show viscosities greater than 100 cP, and this represents the biggest drawback for their use in the extraction processes [42]. Therefore, in order to reduce viscosity and increase extraction efficiency, a certain amount of water was added to the all synthesized NADESs.

The optimization of this dilution process was performed with different ChCl-LA1/water ratios (*v*/*v* = 4/1, 2/1 and 1/1). Higher water contents were not tested because a large excess of water could break the hydrogen bonds between the NADES components and lose the eutectic property of the solvents produced, as observed by others [43,44]. In addition, the increase in temperature results in a) the decrease in the viscosity of the solvent, b) the decrease in the surface tension and c) the increase in the diffusion of analytes into the solvent. However, based on the results published by other authors, it can be concluded that viscosity of DESs/NADESs drops markedly with the temperature increasing up to 313 K (39.85 °C), while further increases in the temperature lead only to minor changes in the solvent properties [45]. Consequently, the selection of the UAE extraction temperature (35 °C) was based on recently published studies [46,47].

**Figure 1:**
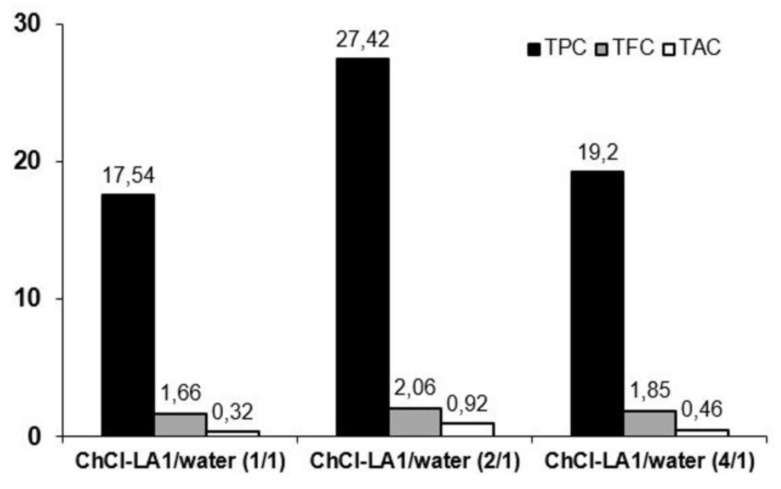
Total phenolic content (TPC), total flavonoid content (TFC) and total monomeric anthocyanin content (TAC) in chokeberry extracts obtained after extraction with different ChCl-LA1 aqueous solutions. TPC was expressed as mg GA g^−1^ DW; TFC was expressed as mg RUT g^−1^ DW; TAC was expressed as mg Cya-3-Glu g^−1^ DW.

Three representative polyphenol indices (dependent variables), namely total phenolic content (TPC), total flavonoid content (TFC) and total monomeric anthocyanin content (TAC), were compared. As shown in Figure 1, the TPC was the highest at ChCl-LA1/water ratio 2/1 (27.42 milligrams of gallic acid equivalents per gram of dry weight (mg GA g^−1^ DW)). A similar pattern was observed for the TFC and TAC, and the highest yields were also achieved using ChCl-LA1/water ratio 2/1 (*v*/*v*), 2.06 milligrams of rutin per gram of dry weight (mg RUT g^−1^ DW) and 0.92 milligrams of cyanidin-3-glucoside equivalents per gram of dry weight (mg Cya-3-Glu g^−1^ DW), respectively. The results published in our earlier work also postulate lactic acid-based NADES with 32.2% water as the most effective extraction media for the isolation of phenolic compounds from dried *Lippia Citriodora* leaves [31].

### 2.2. Selection of the Most Effective NADES for the Extraction of Phenolic Compounds from Dried Aronia

Three groups of HBDs (organic acid, two sugars and urea) were used in combination with choline chloride to produce a total of five different NADESs (Table 1). The extraction efficiency of different phenolic compound classes from chokeberry using synthesized NADESs and 80% MeOH was compared. TPC, TFC and TAC were determined in all extracts obtained and the results are presented in Table 2.

For TFC, ChCl-Fru showed the highest extraction efficiency, followed by ChCl-Glu and 80% MeOH. The lactic acid-based NADESs (ChCl-LA and ChCl-LA1) showed the lowest efficiency in the extraction of total flavonoids from chokeberry (Table 2).

The order of efficiency in the extraction of the TAC from the dried chokeberries was as follows 80%MeOH > ChCl-Fru > ChCl-LA1 > ChCl-Glu > ChCl-LA > ChCl-U (Table 2).

In general, the results confirmed that fructose-based NADES (ChCl-Fru) have the highest capacity for the extraction of various phenolic compounds from chokeberry (33% higher than those with MeOH under the same extraction conditions). Among the lactic acid-based NADESs, ChCl-LA1 showed the higher extraction efficiency; this solvent had the highest molar ratio of lactic acid. The lower viscosity and higher polarity of ChCl-LA1 could lead to a better extraction compared to ChCl-LA [48].

### 2.3. Characterization of Aronia Polyphenols

**Figure 2:**
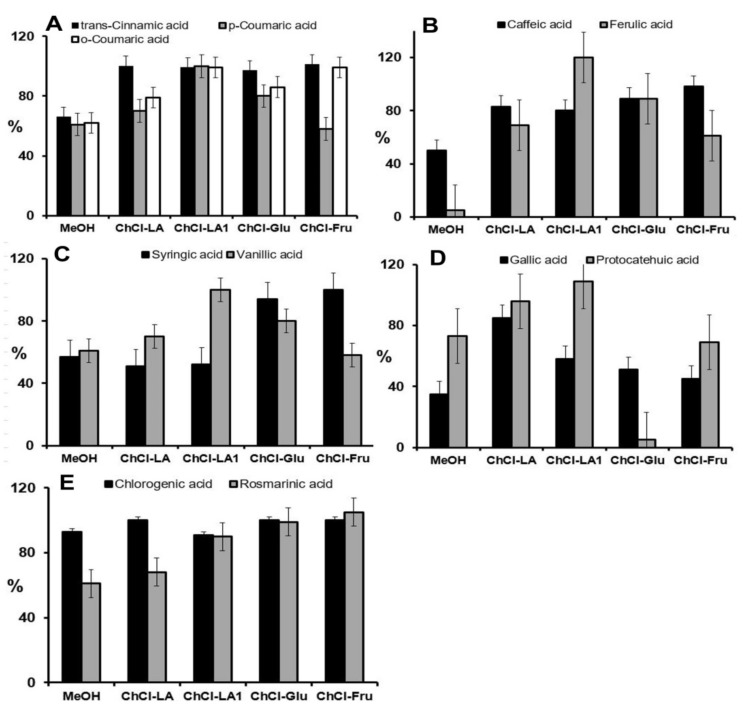
Extraction recoveries of spiked samples (%) for analysed phenolic acids ((**A**) and (**B**)-hydroxycinnamic acids; (**C**) and (**D**)-hydroxybenzoic acids; (**E)**-chlorogenic and rosmarinic acid) obtained after extraction with 80% MeOH and after extraction with different NADESs.

**Figure 3:**
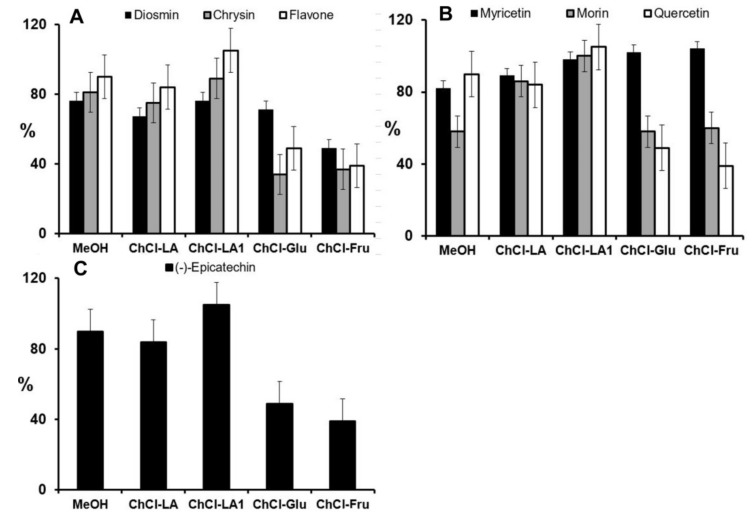
Extraction recoveries of spiked samples (%) for analysed flavonoids ((**A**)-flavones; (**B**)-flavonols; (**C**)-flavanol) obtained after extraction with 80% MeOH and after extraction with different NADESs.

The plant samples showed matrix effects, as the other sample components interfered with the compounds investigated. Therefore, the comparison between spiked and non-spiked samples allowed the best measurement of this effect [49]. The matrix spikes provided the best overall assessment of the accuracy of the developed method. The accuracy after extraction with different NADESs or 80% MeOH was determined by calculating the percentage recovery values. The chokeberry samples were spiked with the known concentrations of the standard mixture (10 mg L^−1^ each compound) and extracted either by methanol or NADESs. The calculated recovery values for each compound are summarized in Figure 2 and Figure 3.

As shown in Figure 2 and Figure 3, the extraction efficiencies in the range from 59.8% to 99.3% were strongly dependent on the type of NADES and the class of phenolic compounds. In general, the high extractability of phenolic compounds with NADESs (compared with an extractability of 80% MeOH) can be attributed to the H-bonding interactions between NADES molecules and phenolic compounds [32].

In addition, the results clearly showed that, in most cases, the NADESs provided a higher extraction efficiency than 80% MeOH for all phenolic acids analysed (Figure 2A–E). This outcome may be related to the fact that various phenolic acids (gallic acid, caffeic acid, *trans*-cinnamic acid and coumaric acids) can be used as hydrogen bond donors for the production of some NADES types [50]. Duan et al. reported on the high extraction capacity of choline chloride-based NADESs for the extraction of various phenolic acids [32]. Our study also confirmed the high extraction capacity of lactic acid-based NADES (ChCl-LA1) for flavonol subclasses of flavonoids such as myricetin, morin and quercetin (Figure 3B).

### 2.4. HPLC-DAD Method Optimisation and Validation

A variety of analytical methods for the separation and detection of phenolic compounds in plant materials and their products have been published, but HPLC with UV-DAD detection remains one of the most commonly used [51].

**Figure 4:**
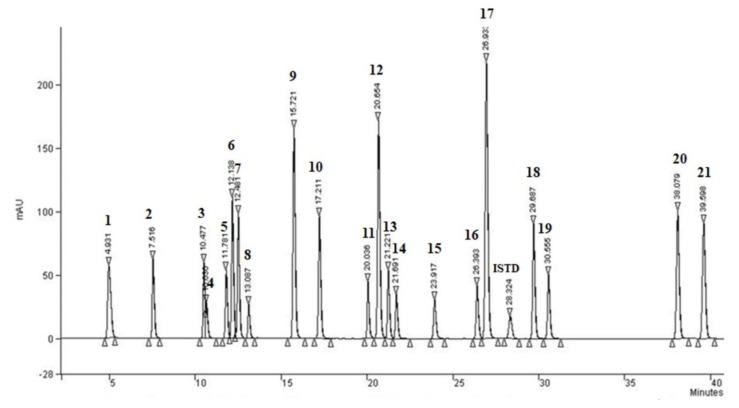
HPLC-DAD chromatogram of standard phenolic compounds mixture (40 mg L^−1^ of each compound) monitored at 280 nm under optimal conditions. The compounds order is listed in Table 3.

The second objective of this study was to optimize the chromatographic separation of investigated phenolic compounds from different classes in a single chromatographic run. Ethyl-o-vanillin was used as the internal standard (ISTD), in all preformed analyses. Ethyl-o-vanillin is a synthetic phenolic compound not found in nature and has similar properties to the target compounds. In the first step of HPLC optimization, different mobile phases consisting of acidified MeOH-water or acetonitrile (ACN)–water mixtures were tested under isocratic conditions for the elution of the compounds. These conditions were tested on mixtures of pure standards of phenolic compounds. The results obtained were broad, completely or partially overlapping peaks with poor resolution. Therefore, the gradient chromatographic method was applied. A series of experiments was performed by changing the gradient conditions. Experiments during the development stage proved that the best chromatographic separation is achieved by choosing acetonitrile instead of methanol as the organic component (solvent A). For acidification (solvent B), the best separation of the analytes between the tested solvents (acetic acid and formic acid, with different percentages) was achieved by using 1% acetic acid. Different flow rates (between 0.5–1.5 mL min^−1^) were tested, and the flow of 1 mL min^−1^ was selected as the optimal rate. At the end of optimization, the best separation of all tested analytes was achieved by the following gradient conditions: 0–1 min 100% (B), 1–5 min 90% (B), 5–45 min 41% (B) and 45–45:01 min 100% (B) within a total run time of 45 min. All compounds were properly detected at a wavelength of 280 nm, although other wavelengths (240, 285 and 320 nm) were also tested. The HPLC-DAD chromatogram of the analysed phenolic compound mixture at a concentration of 40 mg L^−1^, recorded under optimal conditions, is shown in Figure 4.

The developed method was further validated for linearity, precision such as intra-day and inter-day precision, limit of detection (LOD) and limit of quantitation (LOQ). The linearity of the method was tested in the concentration range from LOQ to 50 mg L^−1^ (Table 3). Excellent method linearity was confirmed for almost all compounds investigated (*R*^2^ > 0.9993), with the lowest value calculated for the catechin (*R*^2^ = 0.9967). The linearity of the proposed method was further tested by a one-way ANOVA, which confirmed a significant linear regression and a non-significant deviation from linearity (*p* < 0.05). Intra-day precision (system repeatability) was determined by three consecutive injections of one calibration solution (30 mg L^−1^) on the same day. The relative standard deviations (RSDs) were calculated and varied up to 2.72%. The inter-day precision (intermediate precision) was determined after three repeat injections of standard solutions on three different days. The RSDs ranged between 0.42–4.45%.

The LOD and the LOQ were calculated on the basis of signal-to-noise ratios of 3.3 and 10, respectively. All validation parameters for the compounds investigated, including the average retention times, are summarized in Table 3.

### 2.5. Analysis of Samples

The concentrations of the individual phenolic compounds in the dried chokeberry berries were determined by the developed HPLC-DAD method using the corresponding calibration curves. The results obtained are summarized in Table 4. The results of ChCl-LA1 and ChCl-Fru, as the most promising NADESs for extraction of selected phenolic compounds, were compared to those obtained after extraction with 80% MeOH (control sample). Chlorogenic, protocatechuic and caffeic acid were the main phenolic compounds identified in all extracts. This finding is consistent with the results of other studies on chokeberry (*A. melanocarpa*) [52].

## 3. Materials and Methods

### 3.1. Chemicals

HPLC-grade methanol (MeOH), HPLC-grade acetonitrile (ACN), dimethyl sulfoxide (DMSO), sodium carbonate (Na_2_CO_3_ > 99%), aluminium chloride (AlCl_3_ > 99%), D-(+)-glucose, DL-lactic acid (90%), choline chloride (98%), and standard compounds: ethyl-o-vanillin - ISTD (99%), diosmin (95%), rutin (99%), chrysin (99%), morin hydrate (95%), myricetin ( > 96%), apigenin (97%), (–)-epicatechin (99%), (+)-catechin, vanillic acid (97%), syringic acid (97%), trans-p-coumaric acid (98%), o-coumaric acid (98%), ferulic acid (98%), and rosmarinic acid (97%) were purchased from Sigma-Aldrich (St. Louis, MO, USA). Chlorogenic acid (99%), trans-cinnamic acid (98%), quercetin hydrate (99%) and flavone (99%) were supplied by Acros Organics (Belgium). Protocatehuic acid (99%), caffeic acid (99%) and kaempferol as well as Folin–Ciocalteu phenol reagent (2N), D-(–)-fructose and sodium acetate (CH_3_COONa) were supplied by Merck (Germany). Gallic acid (99%) was purchased from Carlo Erba (Italy). Ultrapure water (resistance above 18 MΩ cm) used was obtained from a Milli-Q water purification system.

### 3.2. Preparation of Standard Solutions and NADESs

Standard stock solutions (1000 mg L^−1^) of the investigated compounds were prepared by accurately weighing 10 mg of each standard into separate 10 mL volumetric flasks and then dissolving them in MeOH. Since diosmin is not completely soluble in MeOH, the stock solution of this standard was prepared by adding 50% DMSO. Working solutions in the concentration range from the limit of quantitation (LOQ, Table 3) to 50 mg L^−1^, were prepared fresh daily by diluting standard stock solutions with ACN.

All chemicals used to produce five different NADESs (Table 1) were dried in a vacuum oven at 60 °C for 24 h before use. HBA choline chloride and the various HBDs (urea, lactic acid, fructose and glucose) were weighted at a certain molar ratio (Table 1), and the mixtures were stirred in sealed bottles at 80 °C until a transparent, colourless liquid formed after 0.5–3 h. The production of sugar-based NADESs required the addition of a certain amount of water (1 mol). All NADESs prepared in this way were further diluted with water to reduce viscosity [50]. The optimization of this dilution was performed on ChCl-LA1. Thus, different ChCl-LA1/water ratios (*v*/*v* = 4/1, 2/1 and 1/1) were tested, and the optimal ChCl-LA1/water ratio was 2/1 (*v*/*v*). The remaining NADESs were then diluted with water in a ratio of 2/1 (*v*/*v*) ratio.

### 3.3. Extraction Procedures

The sample of air-dried chokeberries was purchased from a specialized chokeberry market (Aronija, Slovenia), ground in an electric mixer (Gorenje, Slovenia) for 45 s, packed in glass vessels and stored in a dark place at room temperature until analysed. Exactly 1 g of the ground and homogenized plant sample was weighed into a centrifuge tube, an internal standard (ISTD, ethyl-O-vanillin, 50 mg L^−1^) was spiked and 5 mL of a specific NADES was added [53]. The extraction process was carried out in an ultrasonic bath (Model-LWB 106D, Daihan Labtech Co. Ltd., Korea) for 20 min at 35 °C. After sonication, the sample was centrifuged for 5 min at 7000 rpm (Eppendorf, 5804 R) and the supernatant was transferred with a Pasteur glass pipette into a 10 mL glass flask. The extraction procedure was repeated twice and the supernatants were combined and diluted to exactly 10 mL with the appropriate NADESs.

The efficacy of NADESs for the extraction of phenolic compounds from chokeberry was compared with the efficacy of the most common conventional methanolic extraction (with 80% MeOH) [19]. For this purpose, 1 g of the sample was weighed into a centrifuge tube, an appropriate amount of ISTD was added and the sample was extracted twice using the same extraction procedure as described above. All extractions were performed in duplicate.

The recoveries were obtained by spiking the chokeberry samples with known concentrations of the standard compounds investigated (10 mg L^−1^ each) and extracting them by the method described above (different NADESs or 80% MeOH).

### 3.4. Total Phenolic Content (TPC)

The total phenolic content (TPC) of extracts obtained was determined according to the standard spectrophotometric Folin–Ciocalteu method [54]. A total of 40 μL of properly diluted methanolic or NADES extracts were mixed with 3.160 mL ultrapure water and 200 μL of Folin–Ciocalteu’s phenol reagent. After 6 min, 600 μL of Na_2_CO_3_ (200 g L^−1^) was added. The tubes were allowed to stand for 2 h in a dark place at room temperature. Standard solutions of gallic acid in the concentration range from 50 to 500 mg L^−1^ were prepared in the 80% MeOH. The absorptions of the standards as well as the samples were measured against a blank value at 765 nm using a UV-ViS spectrophotometer (Cary 100 Varian, USA). TPC was expressed as milligrams of gallic acid equivalents per gram of dry weight (mg GAE g^−1^ DW). All samples were analysed in duplicate.

### 3.5. Total Flavonoid Content (TFC)

Total flavonoid content (TFC) was measured by a standard spectrophotometric method [55]. A total of 1.5 mL of pure MeOH was added to 500 μL of properly diluted methanolic or NADES extract and mixed. Afterwards, 0.1 mL of AlCl_3_ (10%), 0.1 mL of CH_3_COONa (1 M) and 2.8 mL of ultrapure water were added. The tubes were left in a dark place at room temperature for 30 min and the absorptions were measured with a UV-ViS spectrophotometer (Cary 100 Varian, USA) against a blank at 415 nm. Standard solutions of rutin in the concentration range from 10 to 100 mg L^−1^ were prepared in the same way. The TFC was expressed as milligrams of rutin per gram of dry weight (mg RUT g^−1^ DW). All samples were analysed in duplicate.

### 3.6. Total Monomeric Anthocyanin Content (TAC)

Total monomeric anthocyanin content (TAC) was determined by the pH differential method [56]. Two suitable aliquots of each extract were diluted with two different buffers (pH = 1 and pH = 4.5). The absorptions of the solutions thus prepared were measured at two different wavelengths: 520 nm and 700 nm. TAC, expressed as mg cyanidin-3-glucoside equivalents per litre (mg Cya-3-Glu L^−1^), was calculated using the following equation:

(1) $$\mathrm{TAC}\;=\frac{A\;\times \;\mathrm{MW}\;\times \;\mathrm{DF}\;\times \;10^{3}}{\varepsilon \;\times \;1}\;$$

where A = (A_520nm_ – A_700nm_)pH _1.0_ – (A_520nm_ – A_700nm_)pH _4.5_; molecular weight (MW) = 449.2 g mol^−1^ for cyanidin-3-glucoside; DF = dilution factor; 1 = path length in cm; ε = 26 900 molar absorptivity coefficient, in L mol^−1^ cm^−1^, for cyanidin-3-glucoside and 10^3^ = conversion factor from g to mg. The final results were expressed as mg of cyanidin-3-glucoside equivalents per gram of dry weight (mg Cya-3-Glu g^−1^ DW). All measurements were carried out in duplicate.

### 3.7. Chromatographic System and Conditions

HPLC analysis was performed on a HPLC-DAD Thermo Scientific Ultra-Mate 3000 system. The separation was performed on an Agilent XDB-C18 chromatography column (150 mm × 4.6 mm I.D., 5 μm particle size). The mobile phase consisted of 100% acetonitrile (solvent A) and 1% aqueous acetic acid solution (solvent B). The gradient program was as follows: 0–1 min 100% (B), 1–5 min 90% (B), 5–45 min 41% (B), 45–45:01 min 100% (B) and re-equilibrium time was 10 min. The injection volume was 10 μL and the flow rate was 1 mL min^−1^ at room temperature. The detection wavelength was set to 280 nm. The identification of phenolic compounds from the samples was determined by comparing their retention times with those of standard compounds measured under the same chromatographic conditions. The quantification was performed using standard calibration curves and an ISTD. Concentrations were expressed as mg of target compound per kilogram of dry weight (mg kg^−1^ DW). All solvents and solutions were filtered through 0.45 μm polytetrafluoroethylene (PTFE) filters before injection.

### 3.8. Validation of the HPLC-DAD Method and Statistical Analysis

The developed method was validated for linearity, intra- and inter-day precision, limit of detection (LOD) and limit of quantitation (LOQ). To determine the linearity, all calibration curves were constructed using an ISTD. The curves were fitted to linear least squares regression (*R*^2^). Precision was evaluated by intra-day and inter-day repeatability, expressed as relative standard deviation in percent (RSD, %). The LOD was determined as the minimal concentration of analyte required to obtain a signal-to-noise ratio of 3.3, and the LOQ was determined as the minimal concentration of analyte required to give a signal-to-noise ratio of 10.

All results in the text and in the tables were expressed as mean values ± standard deviations. Microsoft Excel was used for data preparation and results output. The handling of the statistical data was done with SPSS Statistics (IBM Corp. Released 2013. IBM SPSS Statistics for Windows, Version 22.0. Armonk, NY: IBM Corp.). To determine the significant differences between the extraction recoveries with conventional and different NADESs, a one-way analysis of variance (ANOVA) at the 95% confidence level was also performed.

## 4. Conclusions

The excellent physicochemical properties of natural deep eutectic solvents (NADESs) including adjustable viscosity, high biodegradability, non-toxicity, non-flammability, simple and low cost preparation methods and high extraction power, will encourage application of these solvents in various applications in the future. We have shown, for the first time, that NADESs can be used as green, environmentally friendly and efficient media in the extraction of various phenolic compounds from dried chokeberry fruits. Choline chloride-based NADES as hydrogen bond acceptors in combination with different hydrogen bond donors (organic acid, sugars and urea) were tested. Under the extraction conditions applied, these solvents showed a high extraction efficiency compared to conventional methanolic extraction, thus demonstrating the high potential of the proposed strategy for the extraction of bioactive compounds (gallic acid, protocatehuic acid, chlorogenic acid, vanillic acid, caffeic acid, syringic acid, epicatechin, p-coumaric acid, ferulic acid, quercetin and trans-cinnamic acid) based on NADESs from chokeberry. Due to the dependence of the extraction efficiency on the NADESs composition, specific extractions must be tailored to the desired compound class. Further experiments on extraction from other promising plant materials are ongoing.

## Figures and Tables

**Table 1:** Composition of natural deep eutectic solvents (NADESs).

NADES Composition	Abbreviation	Molar Ratio
Choline chloride:urea	ChCl-U	1:1
Choline chloride:DL-lactic acid	ChCl-LA	1:1
Choline chloride:DL-lactic acid	ChCl-LA1	1:2
Choline chloride: D-(–)-fructose: water	ChCl-Fru	2:1:1
Choline chloride: D-(+)-glucose: water	ChCl-Glu	2:1:1

**Table 2:** Average extraction yields for TPC, TFC and TAC obtained after extraction with different NADESs (NADES/water = 2/1 *v*/*v*) and with 80% MeOH.

Solvent	TPC ^1,2^	TFC ^1,3^	TAC ^1,4^
80% MeOH (control sample)	27.11 ± 1.37 ^b^	3.37 ± 0.25 ^b^	1.25 ± 0.06 ^e^
ChCl-U	17.32 ± 0.61 ^a^	2.50 ± 0.01 ^a^	0.52 ± 0.01 ^a^
ChCl-LA	23.84 ± 1.23 ^b^	1.99 ± 0.12 ^a^	0.57 ± 0.02 ^a^
ChCl-LA1	27.52 ± 0.14 ^b^	2.13 ± 0.10 ^a^	0.93 ± 0.05 ^c^
ChCl-Fru	36.15 ± 3.39 ^c^	4.71 ± 0.33 ^c^	1.01 ± 0.03 ^d^
ChCl-Glu	23.60 ± 0.02 ^b^	3.25 ± 0.08 ^b^	0.83 ± 0.01 ^b^

^1^ Mean values ± standard deviation. ^2^ Total phenolic content (TPC) expressed as mg GA g^−1^ DW. ^3^ Total flavonoid content (TFC) expressed as mg RUT g^−1^ DW. ^4^ Total anthocyanin content (TAC) expressed as mg Cya-3-Glu g^−1^ DW. ^a,b,c,d,e^ Different superscripts for the same response denoted significant differences among solvents tested according to the Student–Newman–Keuls method at *p* < 0.05.

**Table 3:** Validation parameters for HPLC-DAD method: average retention time (*t*_R_); correlation coefficient (*R*^2^); limit of detection (LOD); limit of quantification (LOQ); intra-day and inter-day precision (%RSD).

No.	Compound	*t*_R_ (min)	*R* ^2^	LOD (mg L^−1^)	LOQ (mgL^−1^)	Precision (%RSD)	
						Intra-Day	Inter-Day
1	Gallic acid	4.931	0.9999	0.3	0.9	0.25	2.14
2	Protocatehuic acid	7.516	0.9995	0.2	0.6	0.25	1.14
3	Chlorogenic acid	10.477	0.9993	0.2	0.6	1.19	2.66
4	Catechin	10.630	0.9967	0.4	1.2	1.72	2.34
5	Vanillic acid	11.781	0.9996	0.5	1.5	0.17	1.26
6	Caffeic acid	12.138	0.9996	0.3	0.9	2.49	2.50
7	Syringic acid	12.481	0.9996	0.4	1.2	0.12	1.43
8	(–)-Epicatechin	13.087	0.9998	0.2	0.6	0.25	2.28
9	p-Coumaric acid	15.721	0.9996	0.1	0.3	0.28	1.91
10	Ferulic acid	17.211	0.9996	0.2	0.6	1.18	1.89
11	Diosmin	20.036	0.9997	0.4	1.2	0.58	4.45
12	o-Coumaric acid	20.654	0.9996	0.1	0.3	0.36	2.17
13	Rosmarinic acid	21.221	0.9997	0.3	0.9	0.34	1.23
14	Myricetin	21.691	0,9998	0.3	0.9	1.20	1.99
15	Morin	23.917	0.9996	0.3	0.9	0.42	0.46
16	Quercetin	26.393	0.9994	0.2	0.6	0.62	1.22
17	*trans*-Cinnamic acid	26.933	0.9998	0.1	0.3	0.40	1.47
IS	Ethyl-o-vanillin (ISTD)	28.324	NC	NC	NC	1.01	1.31
18	Apigenin	29.687	0.9999	0.3	0.9	0.83	1.70
19	Kaempferol	30.555	0.9998	0.3	0.9	0.28	1.20
20	Chrysin	38.079	0.9998	0.2	0.6	1.28	2.72
21	Flavone	39.598	0.9995	0.2	0.6	1.16	1.93

NC-not calculated.

**Table 4:** Content of phenolic compound in real dried chokeberry samples. Effect of NADESs ChCl-LA1 and ChCl-Fru. Comparison of the results with those obtained with the traditional method using 80% aqueous MeOH.

No.	Compound	MeOH ^1^	ChCl-LA1 ^1^	ChCl-Fru ^1^
1	Gallic acid	NQ	3.5 ± 0.3	2.6 ± 0.1
2	Protocatehuic acid	109.2 ± 6.4	136.3 ± 8.7	89.1 ± 6.0
3	Chlorogenic acid	1339.8 ± 13.8	2006.9 ± 117.1	1965.0 ± 1.1
4	Catechin	NQ	NQ	NQ
5	Vanillic acid	NQ	29.1 ± 4.2	NQ
6	Caffeic acid	14.2 ± 0.7	17.8 ± 1.1	19.2 ± 0.1
7	Syringic acid	15.8 ± 1.8	9.3 ± 0.1	NQ
8	(–)-Epicatechin	NQ	NQ	NQ
9	p-Coumaric acid	NQ	2.5 ± 0.5	4.6 ± 0.3
10	Ferulic acid	NQ	NQ	NQ
11	Diosmin	NI	NI	NI
12	o-Coumaric acid	NI	NI	NI
13	Rosmarinic acid	NI	NI	NI
14	Myricetin	NI	NI	NI
15	Morin	NI	NI	NI
16	Quercetin	8.6 ± 0.8	28.9 ± 1.5	11.1 ± 1.7
17	*trans*-Cinnamic acid	NQ	NQ	NQ
18	Apigenin	NI	NI	NI
19	Kaempferol	NI	NI	NI
20	Chrysin	NI	NI	NI
21	Flavone	NI	NI	NI

^1^ The results are expressed as mean values ± standard deviations (mg kg^−1^ DW) of two individual measurements. Not identified (NI)-concentration below LOD (see Table 3). Not quantified (NQ)-concentration below LOQ (see Table 3).
